# Abdominal Pain and Dysuria Secondary to Chronic Recreational Ketamine Use: A Case Report on K-cramps

**DOI:** 10.7759/cureus.78422

**Published:** 2025-02-03

**Authors:** Eric Boccio, Jason Haidar, Michael Thiefault, Noy Lutwak, Brian Kohen, Hanan Atia

**Affiliations:** 1 Emergency Medicine, Memorial Healthcare System, Hollywood, USA; 2 Emergency Medicine, Florida International University, Herbert Wertheim College of Medicine, Miami, USA; 3 Pharmacology, Memorial Healthcare System, Pembroke Pines, USA

**Keywords:** abdominal pain, case report, dysuria, k-cramps, ketamine, ketamine toxicity, substance use disorder

## Abstract

Medical and recreational ketamine use is increasing in the United States; however, little is known regarding the side effects associated with chronic, frequent, and high-dose use. The lack of emergency physician awareness regarding ketamine-induced abdominal pain, nausea, vomiting, and dysuria, collectively and colloquially known as K-cramps, results in delayed recognition, underreporting, and inappropriate diagnostic workup and treatment.

A 25-year-old woman with a history of anxiety, asthma, obsessive-compulsive disorder, and chronic high-dose (500-1000 milligrams weekly) ketamine use presented to the emergency department with severe abdominal pain in the right upper quadrant, epigastric, and suprapubic regions, along with nausea, vomiting, and dysuria. Physical exam revealed localized abdominal tenderness but no other significant findings. Lab results including a complete blood count, comprehensive metabolic panel, lipase, beta-human chorionic gonadotropin, and urinalysis were normal. Given the patient’s reported symptoms and frequent, chronic, and high-dose ketamine use, the diagnosis of ketamine-induced abdominal pain and dysuria, or K-cramps, was made. The patient was treated with intravenous fluids, antiemetics, and benzodiazepines. Upon reassessment, all symptoms resolved, and the patient passed an oral challenge. She was discharged with prescriptions for antiemetics and provided with a referral to addiction medicine.

Ketamine-induced abdominal cramping and dysuria, or K-cramps, are scarcely reported in the medical literature but are widely known among chronic users. Treatment in the acute care setting includes a comprehensive history and physical examination, consideration and workup of alternative causes, symptom management, counseling regarding harm reduction, and a referral to addiction medicine resources.

## Introduction

Chronic ketamine use is becoming increasingly prevalent in the United States for both medical and recreational purposes. Ketamine is currently being administered for its analgesic and sedating effects and prescribed to treat numerous mental health conditions such as treatment-resistant depression, post-traumatic stress disorder, and anxiety [1]. It is also self-administered recreationally due to its dissociative effects. Despite its increasing popularity, lower barriers to access, and growing list of indicated and off-label uses, there exists a paucity of information regarding the side effect profile and potential complications of chronic ketamine use and toxicity. Several studies investigating chronic ketamine use for medicinal purposes describe rare occurrences of neurological, gastrointestinal, and urological side effects in subjects [2,3]. Symptoms reported include severe abdominal pain with associated nausea and vomiting and dysuria without increased frequency or urgency. Diagnostic assessments of the hepatobiliary, genitourinary, and gastrointestinal systems are typically unremarkable and unable to determine an underlying cause. Although sparsely mentioned in the medical literature, recurrent and severe abdominal pain associated with chronic ketamine use is well known among frequent users and colloquially referred to as “K-cramps.” We present the case of a 25-year-old woman who presented to the emergency department with two days of severe abdominal pain, nausea and vomiting, and dysuria in the setting of recurrent recreational ketamine use. This case report is one of the first in the medical literature to describe K-cramps formally and describe the clinical presentation, attempt to elucidate the underlying etiology, and provide current recommended therapeutic management options.

## Case presentation

A 25-year-old woman with a past medical history inclusive of anxiety, asthma, postural orthostatic tachycardia syndrome, obsessive-compulsive disorder, and chronic recreational ketamine use with no known surgical history presented to the emergency department for abdominal pain. The patient reported two days of localized abdominal pain in the right upper quadrant, epigastric, and suprapubic regions. The pain was described as severe in severity and cramping in quality. The pain did not radiate to the back, flank, or shoulder. The patient denied positional changes as an exacerbating factor. The patient reported associated nausea and vomiting and was unable to tolerate food, water, and medications per os (PO). The patient stated that she vomited eight times in the two-hour period preceding her arrival at the emergency department; the emesis was not bloody nor bilious. The patient reported her last bowel movement was earlier that morning and appeared grossly normal. The patient denied abdominal distention and reported passing flatus. The patient reported dysuria without increased urinary frequency or urgency. The urine was not reported to be malodorous, and there was no gross hematuria. The patient denied fever and chills. The patient denied vaginal bleeding and reported that her last menstrual period ended three weeks ago. The patient denied alcohol use and reported no prior history of pancreatitis, cholelithiasis, gastritis, gastroparesis, nephrolithiasis, and known gynecologic conditions. The patient stated that she was currently being treated for a urinary tract infection with cephalexin 500 milligrams (mg) PO three times daily which was prescribed five days earlier following a visit to urgent care. The urine culture collected at that time demonstrated no growth to date. The patient stated that she historically had frequently been empirically treated with antibiotics for suspected urinary tract infections, but that corresponding urine cultures were always negative. The patient denied anhedonia, depressed mood, and suicidal and homicidal ideation. The patient reported daily electronic cigarette use and vaped one pod containing approximately 40 mg of nicotine daily. The patient reported weekly ketamine use while attending raves in South Florida during the weekends. The patient self-administered a total of 500-1000 mg intravenously (IV) or intramuscularly on days of use over a three-year period.

Initial vital signs in the emergency department revealed blood pressure of 141/85 millimeters of mercury, pulse of 76 beats per minute, respiratory rate of 18 breaths per minute, peripheral capillary oxygen saturation of 96%, and temperature of 36.6 degrees Celsius, oral. The patient’s measured height and weight were 163 centimeters and 56.2 kilograms, respectively. The patient was alert and oriented to time, place, self, and condition. She appeared uncomfortable due to reported abdominal pain and was standing at the side of the stretcher during initial contact. Physical examination was remarkable for abdominal tenderness with points of maximum tenderness in the right upper quadrant, epigastric, and suprapubic regions. The abdomen was soft and non-distended. No masses were palpated. Murphy’s and Rovsing’s signs were negative. There was no costovertebral angle tenderness bilaterally nor over McBurney’s point. Heart rate and rhythm were normal. There were no rubs, gallops, or murmurs appreciated on cardiac auscultation. Lung sounds were clear to auscultation bilaterally. Pupils were 3-4 millimeters and equal, round, and reactive to light bilaterally. Mucous membranes were moist and there were no tract marks or signs of self-injury across the upper and lower extremities bilaterally. The patient’s mood, affect, and behavior were normal.

Complete blood count with differential, comprehensive metabolic panel, lipase, troponin I, and beta-human chorionic gonadotropin levels were all within normal limits. Urinalysis demonstrated trace leukocyte esterases. Urine microscopy was notable for 4-5 white blood cells, 11-20 red blood cells, many squamous epithelial cells, and rare bacteria. All other components were within normal limits. Upon review of the medical record, urine culture collected at urgent care during a previous visit showed no growth after five days. The electrocardiogram was interpreted as a normal sinus rhythm with a ventricular rate of 82 beats per minute and normal QTc. Radiologic testing was not deemed medically necessary given the patient’s nontoxic appearance, normal vital signs, and unremarkable laboratory results, and, therefore, was not performed. The patient was treated with 4 mg ondansetron IV, 5 mg metoclopramide IV, 25 mg diphenhydramine IV, 1 mg lorazepam IV, and 1 liter lactated ringers IV over 60 minutes. Upon clinical reassessment 120 minutes later, the patient reported no improvement in symptoms. She was given an additional 1 mg lorazepam IV, 5 mg prochlorperazine IV, and 25 mg diphenhydramine IV. One gram ceftriaxone IV was also administered given the patient’s urinary complaints, contaminated urinalysis, and concerns for a false negative result given that the patient had initiated a course of antibiotics two days earlier. Clinical reassessment one hour later revealed that the patient’s symptoms had resolved. The patient successfully tolerated a PO challenge in which she tolerated drinking fluids without vomiting in the emergency department prior to being discharged with prescriptions for 4 mg ondansetron PO every eight hours as needed. The patient was counseled regarding ketamine and nicotine use and provided with an ambulatory referral to addiction medicine. Return precautions were discussed, and the patient reported understanding and agreement with the medical plan.

## Discussion

Ketamine hydrochloride is commonly used for both anesthesia and analgesia in acute medical settings. Recently, its use has become increasingly popular in the therapeutic management of mental health conditions including treatment-resistant depression, post-traumatic stress disorder, and anxiety [1]. Ketamine is commonly used recreationally as its use produces a sedating, euphoric, or dissociative effect for short durations, usually 30-60 minutes. Data from the 2015-2019 National Survey on Drug Use and Health yielded a 0.13% reported use of ketamine in the past year among the 210,392 adults surveyed [4]. Significant risk factors for recreational ketamine use include recent drug use and sexual minority status [4]. Ketamine is classified as a schedule III-controlled substance in the United States due to its reported side effects and potential for addiction and misuse [5].

Ketamine and its metabolites, norketamine and hydroxynorketamine, act on N-methlyl-D-aspartic acid (NMDA), mu-opioid, monoamine, and cholinergic receptors [6]. These interactions are believed to underly the anesthetic, analgesic, and neurocognitive effects of acute ketamine use. Effects on monoamine receptors, specifically serotonin and dopamine, explain ketamine’s role as an antidepressant but have also been associated with gastrointestinal disorders, such as irritable bowel syndrome, functional dyspepsia, and visceral hypersensitivity [6]. The cholinergic effects of ketamine on gastric motility are dose-dependent, with lower doses stimulating motility and higher doses inhibiting it [7]. Similarly, ketamine has dose-dependent effects on NMDA receptors leading to the dysregulation of smooth muscle and increased peripheral sensitization [8]. Ketamine is also known to cause dehydration via reduced saliva production and increased urinary output, and in rare instances, ketamine-induced syndrome of inappropriate antidiuretic hormone secretion and hyponatremia have been reported [9].

Chronic ketamine use has been associated with unusual urinary and hepatobiliary toxicity. Intense upper and lower abdominal pain have been reported following repeated ketamine abuse, the majority of cases to date are urologic. Patients have presented with symptoms ranging from dysuria, frequency, urgency, and painful hematuria [10,11]. Ketamine is extensively metabolized in the liver via the cytochrome P450 enzymes CYP2B6, CYP3A4, and CYP2C9, resulting in the production of the major active metabolites, norketamine, and hydroxynorketamine. Following metabolism, ketamine and its metabolites are predominantly excreted in the urine (approximately 90%). These compounds can be detected in the urine for up to 10 days post-exposure [10,12]. The mechanism leading to ketamine-induced ulcerative cystitis seems to be from direct injury to the urothelial lining caused by ketamine and metabolites. The observed toxicity could be a result of the accumulation of ketamine and its metabolites in the liver and urinary tract, where their concentrations are highest. This is supported by animal studies demonstrating ketamine administration in rats can lead to substantial liver damage, including direct cellular injury, fatty degeneration, and tissue fibrosis [13]. Although short-term ketamine use has rarely been associated with elevated liver enzymes, instances of direct hepatic injury remain infrequent [14,15].

The authors speculate that the mechanism behind the symptoms composing K-cramps may involve a direct toxic effect of ketamine and its metabolites on biliary epithelial cells. Ketamine is a non-competitive NMDA receptor antagonist. By modulating NMDA receptor activity, ketamine affects neuronal excitability and calcium signaling, which are critical for its anesthetic, analgesic, and potentially neurotoxic effects. Ketamine binds to a site within the NMDA receptor channel pore when the receptor is activated, blocking ion flow, including calcium through the receptor. Prolonged or excessive disruption of calcium signaling due to chronic ketamine exposure may contribute to cellular damage, as seen in conditions like ketamine cystitis and liver cell death [16]. Furthermore, ketamine has been shown to dilate cerebral arteries and act as a calcium antagonist. It is conceivable that the effects observed in cerebral arteries extend to the hepatobiliary system, contributing to biliary ductal dilation [17]. Ketamine may also cause hypertonicity of smooth muscle through the alteration of calcium signaling pathways presenting as spasms or cramps clinically [18].

Emergency physicians must consider ketamine-related side effects when evaluating symptoms such as focal right upper quadrant or epigastric abdominal pain or dysuria, especially when both symptoms are present. Although there is no United States Food and Drug Administration-approved treatment for ketamine use disorder, counseling and referral to addiction medicine are recommended. Harm reduction strategies, such as providing resources for safer injection techniques and overdose prevention, are also essential for patients using IV or subcutaneously. Therapeutic management for patients experiencing K-cramps includes discontinuation of ketamine use, fluid and electrolyte repletion for clinical signs of dehydration, antiemetics for nausea and vomiting, histamine type 2 receptor antagonists or proton pump inhibitors for increased gastric secretion, antispasmodic medications such as benzodiazepines or hyoscyamine, dicyclomine, mebeverine, or pinaverium for smooth muscle spasms of the gastrointestinal tract and bladder, and analgesics for acute pain management [19,20].

## Conclusions

The lack of awareness among emergency physicians regarding K-cramps results in delayed recognition, underreporting, inappropriate diagnostic workup and treatment, and insufficient linkage to harm reduction and addiction medicine resources. As both medical and recreational ketamine use continues to increase, further research investigating the etiology of side effects from chronic, frequent, and high-dose use, specifically those related to the hepatobiliary, gastrointestinal, and genitourinary tracts, is warranted.
